# COVID-19 Testing in Sweden During 2020–Split Responsibilities and Multi-Level Challenges

**DOI:** 10.3389/fpubh.2021.754861

**Published:** 2021-11-19

**Authors:** Mio Fredriksson, Anna Hallberg

**Affiliations:** Health Services Research, Department of Public Health and Caring Sciences, Uppsala University, Uppsala, Sweden

**Keywords:** COVID-19 testing, COVID-19 policy, mass testing, population-wide testing, Sweden, government steering, local policy

## Abstract

Sweden's use of soft response measures early in the COVID-19 pandemic received a good deal of international attention. Within Sweden, one of the most debated aspects of the pandemic response has been *COVID-19 testing* and the time it took to increase testing capacity. In this article, the development of and the debate surrounding COVID-19 testing in Sweden during 2020 is described in detail, with a particular focus on the coordination between national and regional actors in the decentralised healthcare system. A qualitative case study was carried out based on qualitative document analysis with a chronological presentation. To understand COVID-19 testing in Sweden, two aspects of its public administration model emerged as particularly important: (i) the large and independent government agencies and (ii) self-governing regions and municipalities. In addition, the responsibility principle in Swedish crisis management was crucial. Overall, the results show that mass testing was a new area for coordination and involved a number of national and regional actors with partly different views on their respective roles, responsibilities and interpretations of the laws and regulations. The description shows the ambiguities in the purpose of testing and the shortcomings in communication and cooperation during the first half of 2020, but after that an increasing consistency among the crucial actors. During the first half of 2020, testing capacity in Sweden was limited and reserved to protect the most vulnerable in society. Because mass testing for viruses is not normally carried out by the 21 self-governing regions responsible for healthcare and communicable disease prevention, and the Public Health Agency of Sweden stated that there was *no medical reason* to test members of the public falling ill with COVID-like symptoms, the responsibility for mass testing fell through the cracks during the first few months of the pandemic. This article thus illustrates problems associated with multi-level governance in healthcare during a crisis and illustrates the discrepancy between the health service's focus on the individual and the public health-oriented work carried out within communicable disease control.

## Introduction

During the first phases of the COVID-19 pandemic, Sweden received international attention for choosing less strict countermeasures compared to many other countries including neighbouring countries such as Norway and Denmark (1) and for relying on voluntary compliance (2). The relative lack of restrictions such as lockdowns or closures of non-essential physical venues illustrates the use of a mitigation strategy with social distancing to protect the elderly and the functioning of the healthcare services, rather than a suppression strategy (3). Within Sweden, one of the most debated aspects of the pandemic response has been *COVID-19 testing* and the time it took to increase testing capacity. In June 2021, when presenting its review of the government's actions, the Swedish parliament's constitutional committee concluded that the government had failed in six cases related to the COVID-19 pandemic. One of these cases was COVID-19 testing, which, according to the committee, started too late and suffered from an unclear division of responsibilities. In particular, the unclear division of responsibilities concerned the state (the government and the government authorities) vis-à-vis the self-governing regions responsible for funding and providing healthcare. This is investigated further in the present article.

**Figure 1:**
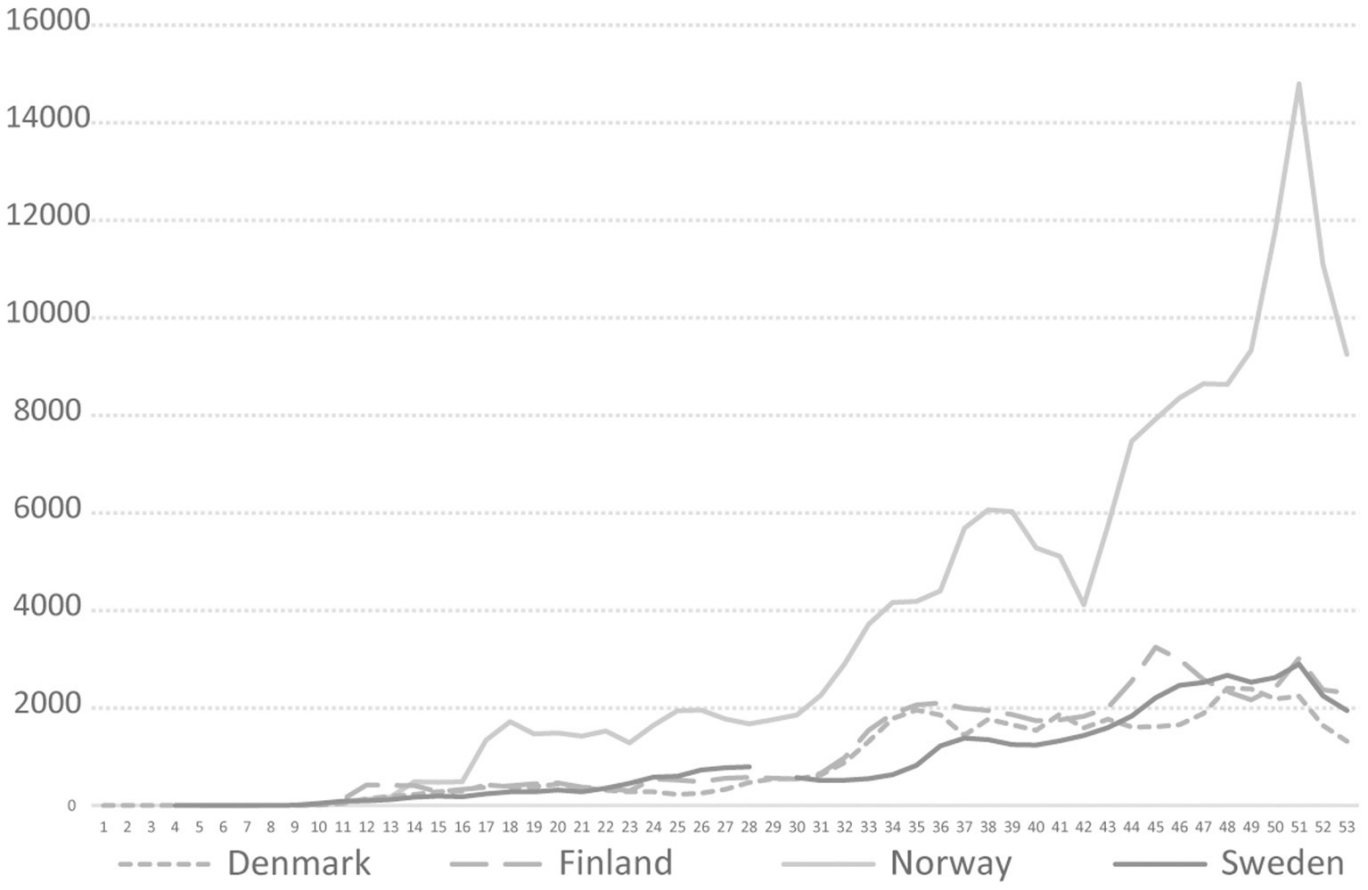
COVID-19 testing rate per 100,000 in four Nordic countries during 2020, Weeks 1–53, 2020. https://www.ecdc.europa.eu/en/publications-data/COVID-19-testing.

**Figure 2:**
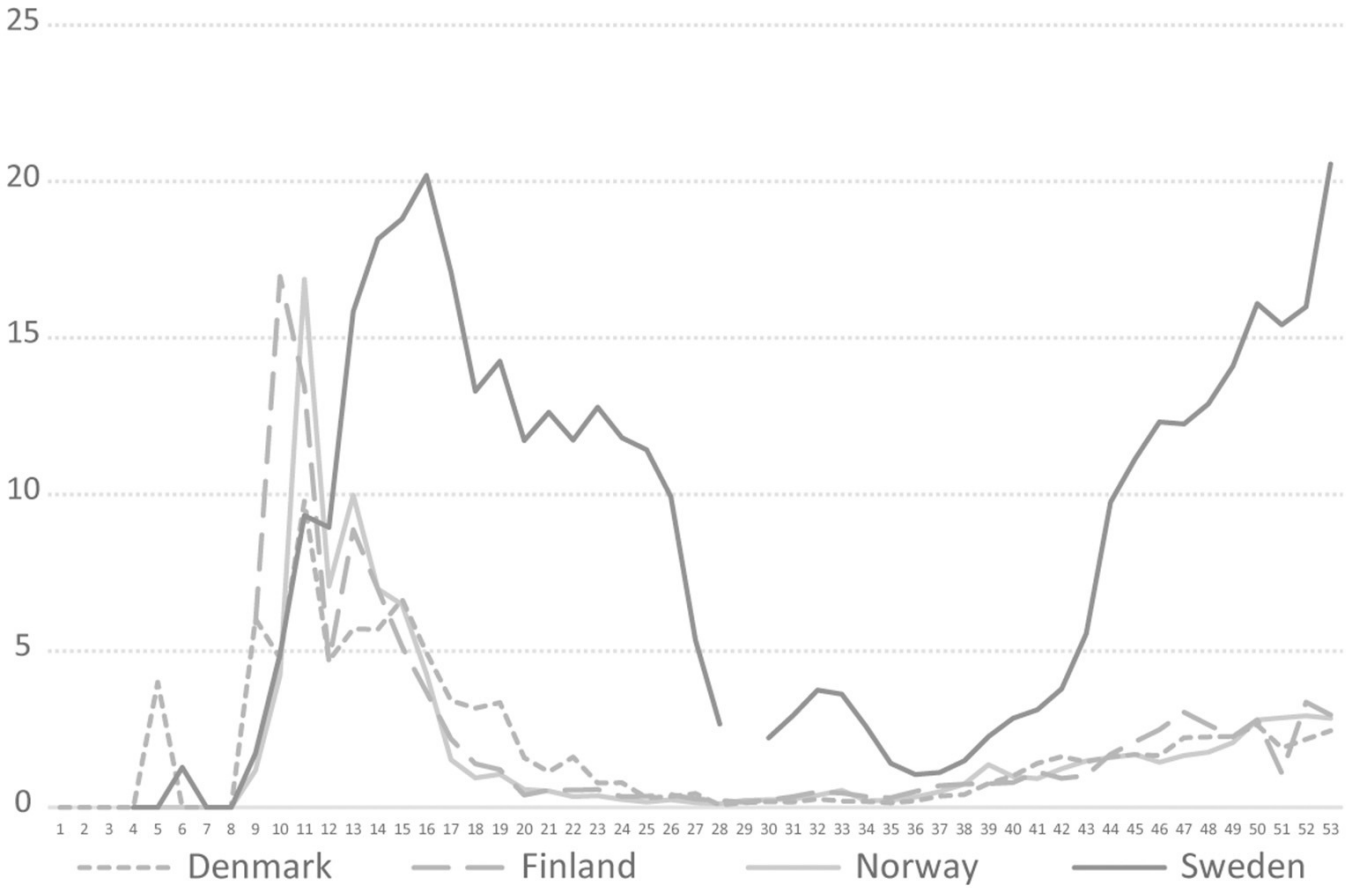
COVID-19 testing positivity rate in four Nordic countries during 2020, Weeks 1–53, 2020. https://www.ecdc.europa.eu/en/publications-data/COVID-19-testing.

**Figure 3:**
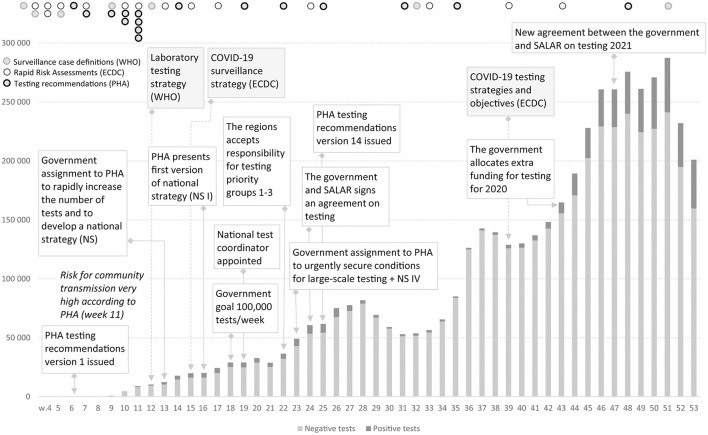
Positive and negative COVID-19 tests in Sweden during 2020, Weeks 1–53, 2020. There are minor variations in the weekly reports on the number of tests taken and number of detected cases. https://www.folkhalsomyndigheten.se/folkhalsorapportering-statistik/statistik-a-o/sjukdomsstatistik/covid-19-veckorapporter/.

The Swedish COVID-19 strategy has been the subject of a few published research studies, so far where some aspects of COVID-19 testing are described (4, 5), and the low level of testing capacity has been linked, for example, to an underestimation of infected persons (6, 7), and mortality in long-term care facilities (3). In international research on COVID-19 testing, mass testing has been suggested as a cornerstone for handling the pandemic (8) and a powerful means to supress the spread instead of, or in addition to, lockdown (9). Among national politicians in Sweden, opinions have differed regarding whether Sweden has tested too little or much in relation to its population size. As shown in Figure 1, in 2020, Sweden tested for COVID-19 far less than Denmark, but roughly in line with Norway and Finland. During the period between July 20 and October 25, Sweden tested fewer people per 100,000 inhabitants than Finland and Norway. For most of 2020, Sweden tested less than Norway. However, looking at the positivity rates, Sweden stood out from mid-March with a much higher number of new confirmed cases per test than other Nordic countries, pointing to a more extensive spread [cp. (10)], and thus less testing in relation to the number of cases (Figure 2). The number of tests per week in Sweden during 2020 is presented in Figure 3, together with some of the most important COVID-19 testing strategy decisions (including some international references).

In this article, the development of and the debate surrounding COVID-19 testing in Sweden during 2020 is described in detail, with a particular focus on the coordination between national and regional actors. This article thus illustrates the problems associated with multi-level governance in healthcare during a crisis and also contributes to understanding one of the most debated aspects of the Swedish pandemic response.

## Methods

### Design

A qualitative case study allowing for in-depth, detailed examination of the particular case of COVID-19 testing in Sweden during 2020 was carried out.

### Data Sources

The sources of data for the in-depth description were publicly available documents (although some not available online) and press material from 2020. From the government, all press releases from the Ministry of Health and Social Affairs with bearing on COVID-19 testing were analysed, as well as the government's assignments to the Public Health Agency of Sweden (PHA). From the PHA, all of their press releases on COVID-19 testing were analysed, as well as the 17 versions of the COVID-19 testing indications and the four versions of the National Strategy for COVID-19 testing. The press releases from the Swedish Association of Local Authorities and Regions (SALAR) were also analysed. In addition, the parliament's constitutional committee examination of the government's handling of the COVID-19 pandemic was used as a source (11), along with weekly statistical reports on COVID-19 from the PHA.

A sample of Swedish national newspapers (*Dagens Nyheter, Svenska Dagbladet, Aftonbladet*, and *Dagens Medicin*) were also analysed based on a search in Retriever Research for “covid test^*^ AND (Lena Hallengren (Minister of Health and Social Affairs) OR Harriet Wallberg (National Coordinator for COVID-19 testing) OR Ulf Kristersson (Opposition leader) OR Anders Tegnell (State Epidemiologist) OR Karin Tegmark Wisell (Head of the Microbiology division at the PHA))”.

### Analysis

The analysis was carried out as a qualitative document analysis (12) with a chronological presentation aiming to describe in detail the process of establishing COVID-19 testing in Sweden. The analysis focussed on finding and describing the main events and arguments, but also on covering different perspectives and actors in relation to the main events. Throughout the chronological description, there is a presentation of the number of COVID-19 tests performed in the country and the number of confirmed cases of COVID-19. There are also some references to published recommendations and/or strategies with bearing on COVID-19 testing by the World Health Organization (WHO) and the European Centre for Disease Prevention and Control (ECDC, an EU agency aimed at strengthening Europe's defences against infectious diseases).

### Context

The analysis took into consideration the specific context of the Swedish multi-level healthcare system and below some crucial contextual information is presented.

#### A Multi-Level System

In Sweden, the responsibility for healthcare is divided between three governing levels: state, regions and municipalities. The *state* is responsible for overall healthcare policy and laws. The *21 regions* are the key actors responsible for funding healthcare (about 80% comes from local taxes and patient fees and 20% from state grants) and for providing healthcare to their residents. The Health and Medical Services Act (2017:30) states that the regions shall provide good healthcare to their residents and work towards their good health (Ch. 8, §1: 2017:30). The *291 municipalities* are responsible for certain types of healthcare related to the long-term care of elderly people and people with disabilities. All of the regions and municipalities are members of the Swedish Association of Local Authorities and Regions (SALAR), which is an employers' organisation that also advocates for local/regional government. SALAR represents the regions and municipalities in discussions and negotiations with the Ministry of Health and Social Affairs. In practise, Swedish healthcare is highly decentralised, and the regions are self-governing with politically elected assemblies, as set down in the Swedish constitution (1974:152), and overall, it can be described as an arrangement with 21 regional health systems.

#### The Government and Government Agencies

The government rules the country by enforcing the parliament's decisions and taking initiatives to develop new laws and amendments to laws. To assist in this work, the government has Government Offices and about *220 government agencies*. Each year the government gives instructions to the government agencies (through a regulation letter) on how to work and how much money they can use. However, the government cannot control how the agencies interpret the laws, and, importantly, in Sweden, the ministers are not allowed to issue orders personally to agencies in their portfolio or to interfere with their day-to-day work (i.e., ban on ministerial rule). Of specific importance for COVID-19 testing has been the *Public Health Agency of Sweden* (PHA), but also to some extent the Swedish Civil Contingencies Agency (MSB), which is responsible for issues concerning civil protection, public safety, emergency management and civil defence, and the National Board of Health and Welfare (NBHW), which the government gave the task of being a national purchaser of, for example, material for COVID-19 tests, and if necessary, redistributing material between the regions (13).

#### Public Health Agency of Sweden

The PHA is an agency that has a national responsibility for public health issues, where provision of knowledge and guidelines is crucial. Their mission is to promote good and equal health, prevent illness and injuries and work for effective communicable disease prevention and control, as well as protecting the public from different types of health threats (14). The PHA has the overall national responsibility for protection of the public against communicable diseases, and from 2014, it also coordinates communicable disease control at the national level.

The agency is led by a director general, and the management group includes the heads of six different divisions and the director general's office (15). At the agency, there is a State epidemiologist and a deputy state epidemiologist, who are tasked coordinating the monitoring and analyses of the development of communicable diseases nationally and internationally and the protection against these diseases. The agency issues regulations (binding clarifications of the law); recommendations (non-binding but building on evidence or best current expert knowledge in areas that may change rapidly due to new or unknown factors); and guidance to healthcare staff to ensure effective disease control. In the case of COVID-19 testing, an important document has been the testing indications (17 versions during 2020, see Supplementary Table 1 for summary)–that is, the recommendations about whom to test for COVID-19 infection.

Another important task of the PHA is carrying out microbiological laboratory analyses and providing expert support in dealing with suspected or established outbreaks of communicable diseases, as well as maintaining the laboratory preparedness needed for effective communicable disease control (14). The agency provides support for quality and methods development at laboratories that carry out analyses of importance for the country's communicable disease control (2021:248, §19). The PHA also provides the government with expert knowledge and information within its portfolio (2021:248, §3). In its *pandemic preparedness plan*, the overarching goals for pandemic responses are to minimise mortality and morbidity in the population and to minimise other negative consequences for individuals and society. According to the PHA, this requires *medical action* (antiviral treatment and vaccinations), *non-medical action* (e.g., social distancing) and *communication*. Broad testing is not mentioned in the pandemic preparedness plan, but it is mentioned that it is the PHA's task to monitor the spread of a virus through laboratory testing. Based on the World Health Organisation's (WHO) guidance for Pandemic Influenza Preparedness and Response (16), the PHA prescribes actions during the activation phase (detailed monitoring of every case and contact tracing), the pandemic phase (where national monitoring data is used to estimate the spread) and post-peak phase (responses can be phased out). There are also two specific preparedness plans for communication and access to medicines during a pandemic (17, 18).

#### Communicable Disease Control

A specific law regulates *communicable disease control* (2004:168), the first chapter of which specifies responsibilities. The PHA is responsible for coordination of communicable disease control at the national level and should take the initiatives necessary to maintain effective control (Ch. 1, §7). Furthermore, each region is responsible for taking necessary communicable disease control measures within its area (§8). The roles and responsibilities of the different authorities during a pandemic specifically is further specified in the pandemic preparedness plan mentioned above. Each region must have an Infection Control Practitioner, a physician responsible for communicable disease control (§9). These have an overall responsibility for the communicable disease control in the region and plan, organise and lead the necessary work (Ch. 6, §1). The law specifies a number of specific tasks [Chap. 6, §2, 1–8)] such as providing information to the public, giving advice to risk groups, supporting healthcare staff and ensuring that caregivers take the necessary measures to prevent the spread of infection.

Furthermore, clinical microbiological laboratories perform analyses on behalf of the healthcare services. There are usually 24 laboratories (the number was extended during the pandemic by the contracting of laboratories that do not perform that kind of analyses during normal conditions), of which the majority operate as part of the regions. Two of the 24 laboratories are private actors contracted by some of the regions (19). All of the 24 regular laboratories are part of a national network initiated by the PHA (together with SALAR) in 2014 and established in 2016. At the national level, there is also a forum for emergency diagnostics.

#### Crisis Management

In Sweden, crisis management is built on collaboration between different agencies, regions and municipalities, as well as between businesses and civil society (20). No specific legislation for overall crisis management is activated, instead the ordinary management structure is kept. Three principles are important: (i) the *responsibility principle*: the one responsible for a service/function under normal conditions is also responsible during a crisis situation; (ii) the *likeness principle*: during a crisis, a service or a function should operate as similarly as possible to normal condition; and (iii) the *proximity principle*: a crisis should be handled where it occurs and by those who are most affected and responsible. It is thus primarily the affected municipality or region that lead an effort. In line with this, there is a specific law that regulates what preparations need to be ensured at the local level (2006:544). Only if the local resources are not enough will it be relevant with government funding (11). Regarding healthcare, the Health and Medical Services Act (2017:30) stipulates that the regions are responsible for planning healthcare so that emergency medical preparedness is maintained (Chap. 7, §2).

## Results

### January–February

On **January 31**, Sweden got its first *confirmed case* of COVID-19 (21). At the request of the PHA, the government decided to include 2019-nCoV in the Communicable Diseases Act by **February 1**, meaning that particular infection control measures could be taken (such as isolation) and that testing became mandatory if assessed as necessary by a physician; contact tracing was also mandated (22). On **February 7**, the PHA issued its first version of recommendations for the conditions under which testing for COVID-19 should take place (at this point labelled “instructions”). Individuals that had been to certain parts of China (or that had been in close contact with an infected person) and that presented with acute illness with fever, cough or dyspnoea, or needed hospital care should be tested (23). While waiting for the test results, patients in need of care would be isolated at infection clinics and people staying at home given information on how to avoid spreading the virus (24). Version 1–6 of the PHA testing recommendations were fairly similar but contained updates on which countries and areas were included in the criteria for testing (2020-02-07; 2020-02-12; 2020-02-24; 2020-02-27; 2020-03-02; 2020-03-06). As a point of reference, in their first Rapid risk assessment (January 17), the ECDC stated that they had developed a guidance document addressing questions on how to identify suspected cases and when to initiate testing (25). Starting on January 11, the WHO continuously updated case definitions for surveillance, i.e., who should be investigated and tested. At the end of January, the WHO's case definition was similar to the first Swedish one (26).

On **February 13**, about 150 COVID-19 tests had been analysed by the PHA's own clinical microbiology laboratory, which had had diagnostics in place since mid-January. Although the PHA still assessed the risk for spread of the virus as low within Sweden, they had engaged in dialogue with laboratories to increase the capacity for *analysing* COVID-19 tests in the regions. At that point, eight laboratories in Sweden, except for the PHA's own laboratories, were about to start analysing COVID-19 tests (27). On **February 26**, about 300 tests had been analysed in Sweden, with two *confirmed cases*, and the PHA still assessed the risk for community transmission as low (28). Two days earlier, on **February 24**, the PHA had recommended that people who had been in some parts of China, Iraq, South Korea and Italy should be aware of symptoms and in such cases should call the national 1177 telephone advise service for an assessment (29). During February and at the beginning of March, there were media reports about people being denied a COVID-19 test although fulfilling the criteria (30, 31), but also about the introduction of COVID-19 testing carried out by specially equipped ambulances in Stockholm (32).

### March

The risk for community transmission within Sweden was updated to the level “moderate” by the PHA on **March 2**, when about 1,000 tests had been analysed, with *14 confirmed cases* (33). The day after, **March 3**, the PHA recommended that people who had visited any of nine regions in Italy and developed symptoms within 14 days, should be tested (this was extended to the Austrian state of Tyrol, including Innsbruck, on March 9) (34). On **March 4**, the PHA recommended that the laboratories analysing COVID-19 tests should investigate the possibility of also routinely looking for COVID-19 in tests from patients with respiratory symptoms without known cause to find undetected cases. Tests from the ordinary sentinel system surveying influenza across the country were also from this point to be analysed by the PHA for COVID-19 (35). This was in line with recommendations from the ECDC on March 2, also presenting five scenarios with options for response. It was acknowledged that different countries may be in different scenarios and that testing approaches needed to be adapted to the situation at local and national levels. However, the ECDC anticipated a rapid increase in the demand for testing and concluded that “countries should consider the roll-out of primary diagnostic testing capacity to local clinical and diagnostic laboratories”(36).

In Sweden, to strengthen COVID-19 preparedness, the government announced on **March 5** that the PHA and the NBHW could use “the resources they needed” and the PHA was assigned to develop their coordination and information efforts towards relevant authorities and actors (37). On **March 10**, the PHA upgraded the risk for community transmission to very high and urged everyone with symptoms of respiratory infection to refrain from social contact (38). The week before, *211 new cases* had been detected (39), and on **March 11** the PHA petitioned the government to limit public gatherings to a maximum of 500 people [the same day as WHO declared COVID-19 as a pandemic (40, 41)]. On **March 13**, Swedish media reported that there was a national shortage of test kits and reagents (42). The same day, the PHA announced that the work of decelerating the spread of COVID-19 in society was entering a new phase and that the new focus was on delaying the spread of infection while simultaneously protecting the oldest and the most fragile against the virus. Thus, the strategy of finding all cases through testing people with symptoms that had been in certain areas abroad was no longer deemed the most effective.

In line with the ECDC recommending that testing approaches should prioritise vulnerable populations, protection of social and healthcare institutions, including staff (if testing capacity was overwhelmed by a large number of tests in countries experiencing localised outbreaks or widespread sustained transmission) (43), the PHA concluded that health services and clinical microbiological laboratories needed to redirect their resources for testing and analysis to where they were needed the most (44), and version 9 of the PHA testing recommendations (now labelled “indications”) were issued (45). The PHA recommended COVID-19 testing for people in need of inpatient hospital care who had become acutely ill with fever or respiratory symptoms, without known cause, and to healthcare and social care staff working with the elderly, falling ill with acute fever and respiratory symptoms. The purpose was to prevent the virus from spreading within healthcare and social care services. The PHA also announced that there was “no specific medical treatment to COVID-19” and “that almost everyone gets a mild illness and recovers after a period of self-care at home.” The agency therefore saw no *medical* reasons to test everyone falling ill with symptoms such as cough, runny nose, fever or anything else that may indicate COVID-19, but could also be another infection. Thus, in version 9 of the PHA testing indications, identifying cases of COVID-19 in society was declared a non-priority (although regions could do this if the regional Infection Control Practitioner assessed it to be relevant from a local epidemiological perspective), which was, however, quickly changed (the day after) to only emphasising which groups should be prioritised (version 10–13). The state epidemiologist said it was of the utmost importance that people were responsible and stayed at home if they were not feeling well and, as a precaution, two days after recovery. The PHA furthermore announced that they would monitor the epidemic through other methods than through extensive testing (44). In line with this, on **March 26**, the PHA announced that they were measuring the occurrence of COVID-19 in the population among randomly selected individuals in Stockholm (46) (about 2.5% had an ongoing infection, see Table 1). At this point, a number of researchers criticised the PHA's recommendations for testing in an open letter to the government and suggested much more extensive testing to be more certain about the spread and to be able to isolate only the individuals who were infected (47). About 2 weeks earlier, on **March 16**, the head of the WHO announced that “Our key message is: test, test, test” and that “social distancing measures and handwashing will not alone extinguish the epidemic”(48). **March 18**, the EU recommendations for testing strategies suggested that “timely and accurate laboratory testing” was “an essential part of the management of COVID-19”. Among other things, it was mentioned that testing helped detecting asymptomatic persons that could spread the virus if not being isolated (49). Similarly, on **March 21**, the WHO's Laboratory testing strategy recommendations recommended countries to scale-up and prepare for a testing surge to, for instance, reduce transmission (although noting that it might be necessary to prioritise who got tested) (50).

**Table 1:** Results from the PHA's population tests to monitor the spread of COVID-19 during 2020.

**Area**	**Test dates**	**Ongoing infection**	**Sample size**
Stockholm	March 26–April 3	2.5%	*n* = 738
Sweden	April 21–24 (a)	0.9%	*n* = 2,571
Stockholm	April 21–24 - subsample of (a)	2.3%	*n* = 679
Sweden	May 25–28 (b)	0.3%	*n* = 2,957
Stockholm	May 25–28 - subsample of (b)	0.7%	*n* = 761
Sweden	August 24–28	No one	*n* = 2,518
Sweden	September 21–25	No one	*n* = 2,461
Sweden	November 30–December 4 (c)	0.7%	*n* = 2,983
Stockholm	November 30–December 4 - subsample of (c)	1.0%	*n* = 790

*Source: PHA*.

As a response to the spread of COVID-19, the Swedish government decided on an amended budget on **March 19**, in which public health and healthcare received an additional 1.5 billion SEK to cover increased costs, such as higher costs for personnel, laboratory analyses and infection control measures (testing and contact tracing were not explicitly mentioned, however) (51). The money could be claimed afterwards and was to be paid out on November 30. In addition, on **the last day of March**, the government assigned the PHA to *rapidly increase the number of COVID-19 tests* (primarily for ongoing infection and later on for antibodies) and to develop a national strategy (NS) for enhancing the capacity to test people for COVID-19 (52). The PHA was tasked with coordinating the work at the national level to expand testing and lead the work of coordinating regions, municipalities, and other actors needed to expand testing while maintaining quality assurance and prioritising resources to healthcare and social care (i.e., making sure that enhanced testing did not crowd out necessary testing of patients and staff) (53). This was to protect the most vulnerable groups in society from the virus. However, another main purpose of the strategy was to reduce negative societal effects from a large loss of staff in healthcare and social care and in other functions of importance for society (52) such as the police and emergency services (54). It was pointed out that necessary staff were probably staying home, although they did not have to if they could be tested. The Minister of Health and Social Affairs, however, pointed out that “it will not be the case that everyone will be able to take a COVID-19 test” (55). In connexion with the government commission, the PHA summoned a number of relevant actors, such as universities and private companies, to meeting on April 1 to ensure diagnostic capacity while maintaining test quality and patient safety (56).

### April–May

On **April 1**, the government decided on another amended budget, in which 1 billion SEK was earmarked for COVID-19 testing (disposed of by the PHA) to reduce the spread of the infection and to avoid unnecessary loss of staff in healthcare and other important societal sectors (57). Up to that point, *3,917 cases of COVID-19* had been confirmed in Sweden (58), and the limit for public gatherings had been lowered to 50 people (59). On **April 10**, the leader of the biggest opposition party criticised the slow pace in building testing capacity in Sweden [about 19,900 tests were taken in Sweden during the period April 6–12 (60), see Figure 1] and argued that the government had to take a clearer leading role, agreeing they in part did so with the assignment to the PHA to develop a national testing strategy (61). At that point, the ECDC concluded that with no indication at EU/EEA level that the peak of the epidemic had been reached, a strong focus should remain on comprehensive testing and surveillance strategies (including contact tracing). If testing capacities were sufficient, all patients meeting the EU case definition should be tested (62).

The PHA presented the first version of the national strategy (NS I) on **April 17** (which was updated April 30 and May 5). By the end of that week, there were *14,577 cases of COVID-19* in Sweden, with about 94,600 tests (63) since the outbreak, and the recommendation from the PHA was that people should stay at home if experiencing symptoms that could indicate COVID-19. It was pointed out by the PHA that NS I was a support in prioritising what groups to test, and the “target image” of NS I was presented as “a nationally secured, sustainable, and robust capacity for testing and diagnostics of COVID-19 within healthcare, eldercare, and institutional care”. The aim was to minimise the spread of the virus within those vulnerable groups. Thereafter, the target image was increased national capacity for testing and analysis of other groups. To achieve this, the PHA estimated that, as a first step, the analytic capacity had to be increased to 150,000 tests per week. It was also pointed out that before the capacity could be expanded, it was necessary to prioritise not crowding out those in greatest need. Priority groups were presented (1–4), as shown in Table 2. About a week later, on April 23, the ECDC concluded that one of the public health objectives was increased testing capacity and that large-scale testing (to detect cases and monitor the spread of the virus combined with contact tracing and isolation measures) was a pivotal criterion of the Joint European Roadmap towards lifting COVID-19 containment measures (64).

**Table 2:** Priority groups presented in the first national strategy for COVID-19 testing.

**Group**	**Description**
1	Patients falling ill with acute infections in need of inpatient care, inpatients at hospitals, individuals belonging to any risk group and residents in care and in institutions.
2	Healthcare and social care staff
3	Individuals having other functions of importance for society; the MSB on May 19 published a list of which functions were classified as important for society during the pandemic (after being advised against publishing it by the PHA because it was not clear enough) (11, 65), as well as a support for employers to identify relevant staff members (66).
4	Other relevant parts of society.

*Source: Nationell strategi för utökad provtagning och laboratorieanalys av covid-19 (NS IV). Since March 12, the testing indications included prioritised groups*.

In NS I, the PHA also suggested how to handle the testing in the regions by presenting different flows for the four priority groups (see Supplementary Table 2 for more details) and pointed out that increased testing required that actors that were normally not contracted by the regions to analyse tests had to assist. A forum for coordination of such actors was also established.

The national strategy was updated on **April 30** to also include serologic testing (i.e., antibody testing; NS II). The PHA meant that, for individuals belonging to a risk group, it could be valuable to know whether they had had COVID-19 and thus be able to lead a more normal life, but staff in healthcare and social care were prioritised (67). On **May 1**, the Minister of Health and Social Affairs said that the goal was to reach 100,000 tests per week by the middle of May (it was reached during the first week of September). This primarily concerned tests for ongoing infection, but the Minister also noted that it was important to build capacity for serologic testing (although there were still problems to solve to ensure testing quality) (68). The Director General of the PHA said that there was now enough laboratory capacity across the country to analyse that number of tests and the Minister emphasised that “the regions had to increase the pace of testing” because fewer tests were performed than the analysis capacity throughout the country allowed. The Minister of Health and Social Affairs also emphasised that the government had been clear with the regions regarding the costs linked to COVID-19 and that they could claim compensation retroactively (69, 70). Between April 27 and May 3, 28,997 individual tests were taken with 3,728 new confirmed cases (13% positive) (71), although the nation-wide laboratory capacity was for about 130,000 analyses during the same period (72).

On **May 8**, the PHA published version 12 of the testing indications: to test those falling ill with symptoms described for COVID-19 within priority groups 1 and 2. It was stated that priority groups 3 and 4 would be included at a later stage and that the work of defining who belonged to priority group 3 (individuals with important functions in society) was under way. It was, however, noted that regional and local adaptations could be made (73). Also, on **May 8**, the government presented the appointment of a test coordinator, who would be placed at the PHA to coordinate the dialogue with the regions regarding large-scale testing for COVID-19 and to increase the pace of testing (74). The chair of SALAR's healthcare delegation commented that several of the regions had worked extensively the past weeks to “gear up to large-scale testing.” The chair pointed to the necessity of developing the proper infrastructure and logistics to increase testing, such as securing transports of taken tests and access to test material (75). **In the middle of May**, the test coordinator's view was that some regions had come far with building testing capacity while others were still only in the beginning stages, and furthermore, the coordinator said that the focus onwards would be to expand testing for priority groups 1–3 (76). However, the test coordinator could not say when testing of priority groups 3 (or 4) would begin or when the 100,000-target would be reached (77).

On **May 19**, the Minister of Health and Social Affairs announced that it would become possible for those ill, but not in need of hospital care (e.g., seeking care at a health centre or other open healthcare facility), to take a COVID-19 test (thus becoming priority group 1) (78). The PHA, however, pointed out that this did not mean that everyone seeking care would be tested, but only if a physician made an assessment that it was relevant from a medical point of view (79). The government also confirmed that it would cover the costs for priority group 3–that is, individuals having functions of importance for society [list on those groups published the previous day (11)]. The national test coordinator explained that there had been a lack of clarity, which had delayed testing, about who belonged to that group and who would fund the testing but said that “Now there are no excuses”(79). About a week later, on **May 26** (in a dialogue between the PHA, the regions and SALAR), the regions made a decision to take responsibility for testing priority groups 1–3 (although this was not properly implemented). This decision was based on the government's repeated assurances that it would fund the testing and that the regions could use private providers and the analysis capacity secured by the PHA at the national level. From this point on, SALAR became more involved in COVID-19 testing, announcing that the regions were quickly building the capacity and competence to secure testing for priority groups 1–3, but that that the responsibility to test priority group 4–that is, other relevant parts of society–was still not clear (80).

The next day, **May 27**, the PHA issued new recommendations for testing (version 13) to include onset of symptoms described for COVID-19 in priority groups 1–3. Priority group 4 was to be included at a later stage, although allowing for regional or local adaptations (81). The same day, the prime minster claimed that the regions had the responsibility for testing all four priority groups, while SALAR replied that the regions and SALAR had not been asked to take this responsibility, and in this case, the purpose and financial compensation had to be discussed (80). The prime minister's press secretary, however, claimed that because the regions have the responsibility for communicable disease control, they have a large role in all testing and contact tracing because but that they could involve other actors (11). On **May 28**, the chair of SALAR's healthcare delegation described the past weeks as “confusing” and explained that the health service works from the point of view of *medical need*, which is why its focus had been on testing patients and staff (priority groups 1 and 2). She further explained that it was unclear for the regions how to handle the other groups and what the purpose of testing these groups was: to get a better understanding of the spread of the virus or to know whether people can go to work or not? (The latter is not a task for the health system to handle according to SALAR) (82).

**At the end of May**, “failed testing” was intensely discussed in the media, and the opposition leader, for instance, blamed the government for the failed testing capacity. He meant that the government had not put its foot down and been clear enough about the responsibilities, funding and timeline (83). When pressured to answer why the Swedish COVID-19 testing had failed, the Minister of Health and Social Affairs answered: “There may by 21 different answers to the question why we have not scaled up our testing capacity. There is not one answer” (84). In line with this, the national test coordinator said that the decentralised system was one reason why the scale-up of testing had been slow, and she also said there had been shortcomings in the regional logistics, for example in the digital systems for referrals and test answers (82). A few days earlier, the Minister of Health and Social Affairs said in a radio interview that “potentially naively” she had thought that when there was analytic capacity for 100,000 tests nationally, and the government had announced it would not be any additional cost for the regions, that the regions would be eager to do the testing rather than finding it hard to build testing capacity (85).

On **May 29**, the PHA published a support document and announced that it was “desirable and justified” to engage in generous testing and contact tracing among elderly and staff in eldercare to discover and prevent the spread of COVID-19 (revised version June 17) (86). In the **last week of May**, about 36,500 individuals were tested and, in total, there had been *38,897 confirmed cases* since the beginning of the outbreak (87).

### June

During the first week of June, nation-wide laboratory capacity was about 140,000 analyses per week, but only about 49,000 tests were analysed (72). On **June 3**, it was announced that the national test coordinator's assignment, which had included to find the bottlenecks, was over (88). On **June 4**, the government proclaimed that it wanted to “see a sharp increase in testing and contact tracing.” It therefore gave the PHA a third assignment, to urgently secure the conditions for *large-scale* COVID-19 testing in collaboration with the regions and the county administrations and allocated 5.9 billion SEK to this enterprise (of which 1 billion was earmarked for contact tracing). For serologic testing, the PHA was supposed to provide a concrete action plan and timeline to urgently build large-scale testing capacity. For ongoing infection (PCR-testing), the PHA was supposed to assist the regions and county administrations with concrete advice for how the capacity could be increased to test *everyone with symptoms*, irrespective of priority group, and to do contact tracing (89).

To specify the tasks and funding procedure, an agreement (a common collaborative form between the government and SALAR) was signed on **June 11**, according to which the government agreed to take on the costs for testing, while the regions agreed to perform the tests in accordance with the recommendations from the PHA (90). It was stated that increased testing was an important aspect for trying to stop the spread of the virus, but that the variation in spread throughout the country required flexibility and regional adaptation regarding how testing was carried out. This was in line with NS IV (which was released by the PHA the previous day, June 10), in which the PHA pointed out that the purpose of testing for ongoing infection varied depending on the pandemic phase and that the regions may be in different pandemic phases at a certain point in time and may thus appropriately have different testing capacities (72). New testing recommendations, version 14, applied from **June 17**: to test individuals presenting symptoms described for COVID-19 (91). This was in line with the ECDC suggesting that an expanded testing strategy aiming for comprehensive testing of all individuals displaying symptoms compatible with COVID-19 was essential. The ECDC recommended that testing efforts were *maximised* and concluded that the obstacles hindering such an approach was now mostly overcome (92). The same overarching message was presented in the following rapid risk assessments (August 10, 2020; September 24, 2020; October 23, 2020). Between June 14 and 20, 59,861 individual tests were analysed with 7,229 new confirmed cases (*52,189 cases in total*) (93).

### July–October

During July and August, the spread of the virus was rather low, and for the last week of July, the number of new cases and people in intensive care with COVID-19 was at the same level as at the end of March (94). On **July 21**, the PHA published guidance on how to assess who is immune to COVID-19 and what that meant for how to have close contact with others (95), and on **July 23**, they provided guidance on contact tracing (96). During **the last week of August**, 85,060 tests were analysed (1.6% positive) and *83,986 cases had been confirmed in total* (97). During that time, there was criticism of the lack of contact tracing in Region Stockholm (98) and a questioning of whether serologic testing had any effect on reducing the spread of the virus (99). On **August 31**, about 10 days after the schools started after summer holiday, the PHA presented new guidance to the regions on testing children and youth for COVID-19. The PHA recommended that children from pre-school to gymnasium be tested if having COVID-19 symptoms, so they could go back to school as soon as possible (100).

On **September 1**, the PHA presented their view on what efforts were needed to reduce the spread of the infection during the year to come. The PHA highlighted staying home when ill, keeping distance and good hand hygiene, but also mentioned *generous testing* and contact tracing (101). By that time, the PHA recommended that people without symptoms, but who had had close contact with an infected person, should avoid close contact with other people. On **September 21**, the government announced that as part of the state budget of 2021, 2 billion SEK would be allocated to continued testing and contact tracing in the regions (102). In the **last week of September**, 128,852 tests were analysed (2.4% positive) and *the total number of confirmed cases was 91,911*. About 2 weeks earlier, the ECDC released a document outlining strategies and objectives for sustainable COVID-19 testing for different epidemiological situations. A number of different objectives were described, e.g., to control transmission (testing all individuals with COVID-19-compatible symptoms as soon as possible after symptom onset) and to mitigate the impact of COVID-19 in healthcare and social-care settings. It was emphasised that “testing strategies should be flexible and rapidly adaptable to change, depending on the local epidemiology, transmission, population dynamics and resources” (103).

From **October 1**, there were new recommendations from the PHA that people sharing a house or living accommodations with an infected person should also get “behavioural instructions,” for example not to go to work (after being contacted by a contact tracer and given more information) and to get a test after 5 days even if not showing any symptoms (104). In an additional revised budget on **October 30**, the government announced an additional 3 billion SEK during 2020 to meet the need for large-scale testing for the remainder of the year (105). During the **last week of October**, 189,301 tests were analysed (9.7% positive) with about 18,500 new confirmed cases and *133,084 confirmed cases in total*. The PHA concluded that there was now an extensive community transmission of COVID-19 (106). About 2 weeks earlier, the PHA had been given the mandate to decide, after consulting the regional Infection Control Practitioner, on local recommendations for restrictions in activities such as travel, restaurant and gym visits and visits to elder care facilities (107). The Minister of Health and Social Affairs maintained her critique from the spring that the regions should have built up the testing capacity faster, which would probably have had positive benefits now (108).

### November–December

On **November 2**, it was reported that Region Stockholm had to pause the system with home testing due to a rapidly increasing queue (16,000 tests) caused by an increasing spread of the virus (109). The week before, there was an increase by 102% in the number of confirmed cases in the country (106). The Minister of Health and Social Affairs indicated that the stop of home-tests in Stockholm was problematic because the state had allocated multi-billion sums to increase testing capacity in the regions (110). The leader of the biggest opposition party said that the Minister of Health and Social Affairs “declared a war against the regions saying that they were responsible for [the failed] testing during the spring,” and that “it was clearly a state responsibility” (110).

On **November 12**, 7 regions reported that they were operating at their maximum capacity for testing, and 16 regions were experiencing increased strain on the system. The Minister of Health and Social Affairs therefore summoned the PHA, the regions and the NBHW to a meeting about testing capacity (where laboratory capacity was identified as a bottleneck) (111). The Minister indicated that large-scale testing was important to combat the spread of the virus and that it was a cause for concern that the regions could not test as much as needed (112). The Section Head of the Division of Health Care at SALAR called the situation “unfortunate,” but also said it was “a snapshot” and that the regions were working hard to increase COVID-19 testing capacity (113). Representatives of some of the regions noted that they had built the testing capacity based on the PHA's predictions for the autumn–which was cluster spread–and that they would have prepared differently if they had known there would be a second wave of infection (114). Again, leaders of the opposition parties criticised the government's handling of COVID-19 testing and demanded that the government present a plan for increasing testing capacity (115). In the week of **November 9–15**, 254,295 tests were analysed (12.9% positive) with about 31,400 new cases, an increase with 24% from the week before (116).

On **November 18**, the media reported that about half of the regions were operating at their maximum capacity for testing, due to (among other causes) lack of testing material, staff and laboratory capacity. A few regions bought analysis capacity from abroad and thought the national coordination of laboratory capacity was insufficient, and some regions indicated that the PHA had failed in securing access to national analysis capacity (which they had agreed to deliver in the agreement from June 11). The PHA replied that they would order more tests from the laboratories they had contracted, but that a “gradual scaling up of capacity in line with the regions' needs had been assessed as the most responsible management” because analytic capacity is costly. The PHA emphasised that the main responsibility for testing was placed with the regions (117). The PHA announced that they would support the regions in enhancing analytic capacity even further (about half of all analyses were at that time carried out by laboratories contracted by the PHA), for instance by contracting with more laboratories that the regions could use and ensuring that the contracted laboratories increased their staffing (118). This was linked to an extension of the assignment initiated June 4 for the PHA to secure the conditions for large-scale testing. The extended assignment included measures to meet the need for large-scale testing that had occurred because of the rapid spread of the virus and measures to secure preparedness for a scenario of extended and even higher spread (119). Thus, on **November 19**, there was a new agreement between the government and SALAR concerning COVID-19 testing during 2021, which in large part was a continuation of the agreement for 2020 (from June 11). For example, the government agreed to take on the costs for all PCR-tests and the regions agreed to prioritise PCR-tests before serological tests in case of capacity deficiencies. A representative of the PHA pointed out that testing capacity in the regions was currently strained and called on people not to be tested without symptoms or guidance from a physician (120). Furthermore, some debaters suggested that it was irresponsible to expand testing without any limits, because it could crowd out other important tests the regions needed to carry out, such as cervical screening tests (121). **During the period November 16–22**, the number of confirmed cases increased by 2.1% from the week before (31,975 new cases) and 260,710 individuals were tested (11.9% positive). In total, there had been *221,780 confirmed cases* since the outbreak (122).

On November 27, the PHA presented new guidance on rapid tests that had previously been regarded too uncertain, but was now assessed to be a complement to the more certain, but more time consuming PCR-tests (123, 124). The PHA and the government authorised an increased use of rapid tests in some contexts such as eldercare homes (125). The increased use of rapid tests was extended to healthcare and social care in an agreement between the government and SALAR on December 18, with the aim of finding asymptomatic infected staff members (126). The agreement meant that the government would fund the use of rapid tests and the regions would carry them out (127). The week before Christmas, 287,428 individuals were tested and the number of new confirmed cases was 46,210 (16% positive) (128). The last weekly report from the PHA for 2020 summarised a total number of *462,470 confirmed COVID-19 cases during the year*, with 8,443 deaths among people with confirmed COVID-19 (129).

## Discussion

As the detailed description illustrates, the build-up of COVID-19 testing in Sweden during 2020 was a highly complex enterprise with a number of national and regional actors involved with partly different views on their respective roles, responsibilities and interpretations of the laws and regulations. An important aspect of the process is that mass testing was a new area for coordination, because it was an unplanned feature for a pandemic response. In general, the description shows ambiguities in the purpose of testing and shortcomings in communication and cooperation during the first half of 2020, but after that an increasing consistency among the crucial actors regarding COVID-19 testing. In the Swedish debate surrounding COVID-19 testing, there was a lack of explicit reference to WHO and ECDC guidance.

During 2020, the Swedish Agency for Public Management concluded that three aspects of the Swedish public administration model constitute challenges for integrated action during a crisis such as the COVID-19 pandemic, when decisions need to be made quickly and with incomplete information, but with the usual governance tools. Two of these aspects are crucial to understanding the events surrounding COVID-19 testing during the first year of the pandemic: (i) the large and independent government agencies and (ii) self-governing regions and municipalities. Linked to both these aspects is the responsibility principle (130).

### Large and Independent Government Agencies

Sweden has comparatively large government agencies that have extensive independence even if they are subject to the government. The agencies have a delegated responsibility for handling issues within their areas of responsibility and are important in handling a crisis such as the COVID-19 pandemic, without needing to wait for instructions from the government to act (130). In the case of COVID-19 testing, this is illustrated by the fact that there was no formal government steering of the PHA during the two months after the first confirmed case. The first government assignment to the PHA came at the end of March: to increase the number of tests and to develop a national COVID-19 testing strategy. However, the government was not satisfied with the rate of increase in the number of tests in the following one and a half months after the national strategy was published on April 17, and therefore increased its steering through a new assignment to the PHA on June 4: to secure the conditions for large-scale COVID-19 testing for *anyone with symptoms*. In addition, there was increased government involvement, which was reflected, for example, in the number of times testing was addressed in the media by the Minister of Health and Social Affairs. The government also appointed a national test coordinator in May, to be placed at the PHA to coordinate the dialogue with the regions regarding large-scale testing for COVID-19 and to increase the pace of testing. The government and the PHA thus came to work partly in parallel, at times sending mixed messages. This suggests the need for increased coordination among actors at the national level and potentially also for more national steering and coordination in the case of COVID-19 testing than what was possible through the PHA.

Discussing the role of the PHA in COVID-19 testing, it is important to notice the distinction between *taking* COVID-19 tests and *analysing* them, because the PHA's responsibility for these two tasks differs. Regarding analysis, one of the agency's tasks is to provide support for quality and methods development at laboratories analysing COVID-19 tests (and other types of tests important for communicable disease control) and to maintain the required laboratory preparedness (131). Their responsibility to the regions is further clarified in the agreement between the regions and the PHA, which specifies that the agency is responsible for “securing that the health services has access to the analysis capacity needed for the country's infection control” (132). The detailed description illustrates that the PHA was much more involved in securing and enhancing analysis capacity across the country during 2020 than in the logistics for taking tests (much of the global guidance also focused on analysis capacity). For example, according to the agreement between the government and SALAR from June 11, the PHA was to secure analytic capacity and the agency thus contracted and validated new laboratories and coordinated their efforts with the regions' needs during the autumn (although there was a shortage of analysis capacity during November due to the second wave). In line with this division of responsibility for different aspects of testing, there was a discrepancy between tests taken and the capacity for analysing those tests for several months during the first half of 2020. For example, at the end of May, the analysis capacity had quickly gone up to about 135,000 tests/week, but that many tests were not taken in the regions until the first week of September.

The responsibility of the PHA for *taking* COVID-19 tests–particularly for enhancing the capacity to take tests–was less clear, because tests are taken by the health care services in the regions, and this is generally seen as a regional responsibility (although, as will be discussed below, this too was a question of dispute in the case of COVID-19 testing). Importantly, however, in its capacity as an expert agency, the PHA issued support in the form of recommendations for *whom to test for COVID-19* (17 versions during 2020). Although the PHA's recommendations are non-binding, and the document with testing indications from version 7 (March 9) included some formulations about the possibility for local and regional adaptations depending on the epidemiological situation, these recommendations included a clear order of priority for testing that did not encompass staff in essential services until May 27 (priority group 3) and the public until June 17 (priority group 4). The first five versions were also labelled *instructions* to the healthcare services about whom to test (later on the terminology testing *indications* was used) and included exposure factors and clinical indications. These recommendations were issued during a time when relatively little was known about COVID-19, such as how to stop the spread and how best to treat people with COVID-19, as well as when it was difficult for the regions to get an overview of the best response in the midst of rapid knowledge development. In line with this, the national coordinator for COVID-19 testing said in the March 25, 2021, hearing by the parliament's constitutional committee that it was clear the regions followed the testing indications from the PHA, which, during May when the slow pace in increasing testing capacity was heavily criticised by the political opposition, for example, was to test priority groups 1 and 2 (11). The Moderate party chairperson of SALAR explained that one reason why it took so long for the regions to start COVID-19 testing for the public in Sweden was that they followed the recommendations from the PHA. He furthermore explained that the regions had not been willing to scale-up to mass testing before they felt they had support from the national authorities (11). Indeed, it is not unlikely that the PHA's testing indications affected the pace of the build-up of testing capacity. It is, however, important to note that this was also intentional to a certain extent. The recommendations served the purpose of securing that the right individuals were tested and that testing was of high quality during a time when high pressure was put on the regions to expand their testing despite a lack of capacity to do so in some cases. Similarly to this, the WHO and ECDC strategies also pointed to the need to prioritise who was tested if the number of suspected cases exceeded the available testing capacity, however continuously pointing to the need to scale-up testing capacity to manage COVID-19.

Linked to this was also the PHA's communication regarding the general *purpose* of COVID-19 testing. The national coordinator's view was that the PHA communicated that testing was not a strategy to suppress the spread of the disease, but to protect the healthcare system (patients and staff) and get people back to work (11). Therefore, in the coordinator's view, there were no incentives for the regions to build up testing capacity and capacity for contact tracing quickly (11). From March 13 to June 17, the PHA communicated that the purpose of COVID-19 testing was to protect vulnerable groups by preventing the virus from spreading within the healthcare and social care services, and that there were no *medical* reasons to test members of the public falling ill with COVID-like symptoms (in line with the PHA's pandemic preparedness plan linked to the WHO pandemic phases, in which mass testing is not mentioned during the pandemic phase). Thus, during the first half of 2020, the efforts in the regions were directed more towards ensuring that the existing testing capacity was reserved for vulnerable groups and healthcare and social care staff than towards enhancing general testing capacity. This was a balancing act between quickly expanding testing while simultaneously avoiding negative side effects such as displacement, which illustrates the discrepancy between the health care service's focus on the individual and the public health-oriented work carried out within communicable disease control. In September, however, the PHA saw contact tracing in combination with generous testing as one of the most important efforts to reduce the spread of COVID-19 and to minimise the number of sick and dead in the population (133).

### Self-Governing Regions and Municipalities

In Sweden, a large share of public services such as healthcare and elder care is the responsibility of the self-governing regions and municipalities. Although the *responsibility principle* implies that the **one** responsible for a service/function under normal conditions is also responsible during a crisis situation, many of Sweden's national pandemic responses, such as COVID-19 testing, required measures to be taken within the self-governing regions and municipalities (130). Although self-governing, the government can indirectly steer the regions, for example through assignments to the government agencies to coordinate the regions' work and through targeted government grants. This is a “softer” form of steering than, for example, legislation, which also implies that it is more uncertain what the actual response and result will be come. For COVID-19 testing, the government earmarked funding for testing and contact tracing during the spring of 2020 as well as during the autumn of 2020. In total, the government added substantial resources to increase testing, both to develop testing capacity during the spring and to expand it further during the autumn when the second wave hit Sweden. The government stated that testing would not fail because of a lack of funding.

The government also commissioned the PHA to coordinate the regions' work. As shown above, however, the agency's authority is greater for the analysis component of testing, while it is more restricted regarding logistics related to taking tests. The government can also reach out to SALAR (the organisation representing the regions and municipalities) to communicate and negotiate with the regions, which is a form of soft-law governance that is common in Sweden (130) and usually involves both some economic stimuli and coordination efforts. However, according to SALAR, the dialogue between the government and SALAR regarding COVID-19 testing was not sufficient during the first months of the pandemic. Furthermore, SALAR considered that the government could have consulted SALAR to a much higher extent, which would have been more efficient and would have made the regions more prepared for scaling-up. For example, the regions were not consulted by the government before giving the PHA the assignment to extend testing capacity on March 31. In the parliament's constitutional committee hearing on April 9, 2021, the Minister of Health and Social Affairs said that an earlier agreement with SALAR about large-scale testing would probably have led to a more rapid increase in testing capacity. Although the government allocated substantial additional resources for COVID-19 responses in the regions in general, and to testing and contact tracing more specifically, it was initially handled outside the usual agreement structure between the government and SALAR, which caused uncertainty and a lack of clarity for the regions about the level of additional funding and how they would receive it. This was identified as one reason why there was a delay in the increase in testing capacity (11).

Lack of clarity about the responsibility for COVID-19 testing during the first half of 2020 also delayed the increase in testing capacity (11). There were different interpretations of the responsibility for testing the different priority groups. According to the Communicable Diseases Act, the PHA is responsible for coordination of communicable disease control at the national level and should take the initiatives necessary to maintain effective communicable disease control (Ch. 1, §7), while each region is responsible for taking necessary communicable disease control measures within the region's area (Ch. 1, §8). The government's standpoint was that all COVID-19 testing (all priority groups) was the regions' responsibility, although they acknowledged that additional funding was needed for the regions to be able to execute the task and thus provided additional funding (11). The Director General of the PHA also stressed that it was the regions' task to test all groups (11). SALAR, however, had a different interpretation of the regions' responsibilities for testing. They did not question the regions' responsibility for testing priority group 1 (inpatients and residents in healthcare and social care institutions) and priority group 2 (healthcare and social care staff), but just for priority groups 3 and 4. This was in part, again, related to the purpose of testing and the different purposes for testing these groups as formulated by the PHA. Based on the PHA's national strategy for COVID-19 testing from April 17, where it was pointed out that there was no *medical* need for a person from priority group 4 (the public) to know whether they had COVID-19, but rather economic and societal benefits if people could return to work (for group 3 as well), SALAR made the assessment that it was not the regions' responsibility to test priority groups 3 and 4 for COVID-19. To get people back to work was not seen as a task that was covered by the regions' responsibility for taking necessary communicable disease control measures within their areas. Up until the end of May, this interpretation was enhanced by the PHA's testing indications, which stated that priority groups 3 and 4 would be included at a later stage. During the same period, however, the Minster of Health and Social Affairs tried to put pressure on the regions to expand testing capacity by presenting a target of 100,000 tests per week to be reached by the end of May and criticising the regions for being slow in building testing capacity.

Throughout 2020, the political opposition criticised the government for not taking control of the testing situation and also for criticising the regions for being slow. Yet, in the Swedish system, it is unclear how far the state's responsibility to create good conditions for regions and municipalities extends (130). Based on the events linked to COVID-19 testing, it can be concluded that decentralised health care systems such as Sweden's may not be entirely effective during a crisis when quick and coordinated action is needed (unless there are well-functioning coordination mechanisms in place). The benefits of this system, such as local democratic practises and services tailored to suit the local population, may be more visible under other conditions. Simultaneously, the importance of place-based approaches in the response to the pandemic has grown, and the importance of leaving room for local initiatives and experimentation in both centralised and decentralised systems has been pointed out (134). It should also be noted that testing was only one of several areas subject to coordination during the pandemic and was surrounded by much complexity and uncertainties throughout 2020. For other areas for coordination, such as securing intensive care capacity, the coordination process might have functioned differently. The problem of quickly building testing capacity has, however, added fuel to the ever-present discussion on whether to reduce the number of Swedish regions or to nationalise the healthcare system. It has also been argued that there should be a change in the constitutional law so that the regions and municipalities could be subordinated to the government in the event of a national crisis to enhance governmental capacity (135). In his article about the first eight months of Sweden's COVID-19 strategy, Ludvigsson (6) argued that a lack of testing in Sweden led to an underestimation of cases. In our article, the purpose was not to discuss how the Swedish COVID-19 testing system affected the spread of the disease, illness in the population or deaths among people with confirmed COVID-19, but this would, however, be a relevant topic for future study, drawing on this description.

### Limitations

There are some limitations to this study. Although the description is detailed, it has not been possible to include all events or discussions that may be linked to COVID-19 testing in Sweden during 2020. However, all of the most important events and discussions are covered. Furthermore, this study only covered events, discussions and documents published at the national level by national actors. Even more nuanced descriptions of the build-up of COVID-19 testing in Sweden could be made by analysing regional documents, debates and media coverage. In addition, this study only covered documents and media articles that are publicly available, which means that internal meetings and informal conversations or negotiations were not covered. Informal contacts have been identified as important for the government to clarify their governance. Lastly, other perspectives on COVID-19 testing could be unravelled in interviews with representatives from the regions, SALAR and government agencies such as the PHA, and such a study is under way.

## Conclusion

During the first half of 2020, COVID-19 testing capacity in Sweden was limited in relation to the spread of the virus and reserved for inpatients, people in institutional care and healthcare and social care staff in order to protect the most vulnerable. The move to mass testing proved to be complex in part due to the responsibility principle in Swedish crisis management, which makes clear that the responsibility of a specific area is to remain with the actor that normally manages it. Because mass testing for viruses is not something that is normally carried out by the 21 self-governing regions responsible for healthcare and communicable disease prevention, and the PHA stated that there was no medical reason to test members of the public falling ill with COVID-like symptoms, the responsibility for mass testing fell through the cracks during the first few months of the pandemic. Through increased government steering and increased communication between the government, the PHA and the regions (SALAR), the question marks around roles and responsibilities for testing were straightened out by the end of spring 2020, and testing capacity was much increased during the autumn. Until September, the capacity to analyse COVID-19 tests–in which the PHA had a pronounced role–was higher than the regions' capacity to take the tests.

## Data Availability Statement

Publicly available data were analysed in this study. This data can be found here: https://www.regeringen.se/sveriges-regering/socialdepartementet/; https://www.folkhalsomyndigheten.se/; https://skr.se/skr/tjanster/press.408.html; https://www.ecdc.europa.eu/en/publications-data/covid-19-testing.

## Author Contributions

MF planned the study, carried out the majority of the analyses of the empirical material, and drafted the article. AH was involved in collecting the empirical material, critical review of the analyses and the discussion, and was involved in finalising the article. All authors contributed to the article and approved the submitted version.

## Funding

This study was funded by the Swedish Research Council for Health, Working Life and Welfare (2018-01578).

## Conflict of Interest

The authors declare that the research was conducted in the absence of any commercial or financial relationships that could be construed as a potential conflict of interest.

## Publisher's Note

All claims expressed in this article are solely those of the authors and do not necessarily represent those of their affiliated organizations, or those of the publisher, the editors and the reviewers. Any product that may be evaluated in this article, or claim that may be made by its manufacturer, is not guaranteed or endorsed by the publisher.
