# Supplementary material for: COVID-19 Testing in Sweden During 2020–Split Responsibilities and Multi-Level Challenges
Source: Front Public Health. 2021 Nov 19;9:754861. doi: 10.3389/fpubh.2021.754861 (PMC8639858; doi:10.3389/fpubh.2021.754861)
Supplement: Supplementary file 1 [file Data_Sheet_1.PDF]

Table S1. Summary of testing recommendations from the Public Health Agency of Sweden for COVID-19 during 2020

Version and date	Overall purpose	Specification of testing	Local/regional adaptations
(1) 2020-02-07<br>(2) 2020-02-12<br>(3) 2020-02-24<br>(4) 2020-02-27<br>(5) 2020-03-02	An instruction for the healthcare services under which conditions testing for COVID-19 should take place<br><br>The purpose of testing is to 1) identify cases of COVID-19 where there is a risk of societal or healthcare-related spread of infection, and 2) map out infection chains by contract tracing and increase knowledge about transmission.	Individuals who meet two types of criteria should be tested.<br><br>1) Epidemiology factors (exposures such as having been in certain areas abroad or having been in close contact with someone with confirmed COVID-19) **AND**<br>2) Clinical indications such as acute illness with fever and cough without known cause.	
(6) 2020-03-06	Testing indication for potential COVID-19<br><br>The purpose of testing is to 1) identify cases of COVID-19 where there is a risk of societal or healthcare-related spread of infection.	Individuals who meet two types of criteria should be tested.<br><br>1) Epidemiology factors (exposures such as having been in certain areas abroad or having been in close contact with someone with confirmed COVID-19) **AND**<br>2) Clinical indications such as acute illness with fever and cough without known cause.	-
(7) 2020-03-09<br>(8) 2020-03-10	Testing indication for potential COVID-19<br><br>The purpose of testing is to 1) identify cases of COVID-19 where there is a risk of societal or healthcare-related spread of infection.	Individuals who meet two types of criteria should be tested.<br><br>1) Epidemiology factors (exposures such as having been in certain areas abroad or having been in close contact with someone with confirmed COVID-19) **AND**<br>2) Clinical indications such as acute illness with fever and cough without known cause.<br><br>3) Testing is also indicated in the case of acute lower respiratory tract infection with the need for hospitalization regardless of risk exposure [a]	[a] Regional and local adaptations are handled in each region where the respective Infection Control Unit in collaboration with the infection clinic/s and clinical microbiology leads and coordinates appropriate management of COVID-19 so that adaptation to what is most appropriate at the regional level is considered.

(9) 2020-03-12	Testing strategy for COVID-19.<br>Focuses on identifying cases in healthcare in order to protect the most vulnerable groups.<br><br>Suspected cases in society of individuals with good general condition and without the need for medical care are handled primarily through isolation in the home and social distancing.	Category 1A – priority [b]<br>Patients in need of inpatient care in hospital (+ clinical indications)<br><br>Category 1B – priority [b]<br>Staff in healthcare and elderly care at known COVID-19 exposure (+ clinical indications)<br><br>Category 2 - non-priority [b]<br>Identification of cases of COVID-19 in society (exposure + clinical indications)	In regions where the Infection Control Practitioner, based on the regional epidemiological situation, deems it relevant to test potential cases in society in individuals with good general condition, regional guidelines are referred to.<br><br>[b] Regional and local adaptations are coordinated by Infection Control Practitioners based on what is most appropriate based on the current epidemiological situation and regional and local structures.
(10) 2020-03-13	Testing strategy that focuses on identifying cases in healthcare in order to protect the most vulnerable groups.	Category 1A - priority<br>Patients in need of inpatient care in hospital (+ clinical indications)<br><br>Category 1B<br>Staff in healthcare and elderly care based on what is most appropriate based on current resource needs (+ clinical indications)	Regional and local adaptations are coordinated by Infection Control Practitioners based on what is most appropriate based on the current epidemiological situation and regional and local structures.
(11) 2020-04-01	Testing indication for COVID-19<br><br>Focuses on identifying cases in healthcare in order to protect the most vulnerable groups.	Category 1 - priority<br>Patients in need of inpatient health care or social care and people already inpatient at hospitals and care homes (+clinical indications)<br><br>Category 2A<br>Staff in healthcare and social care based on what is most appropriate based on current resource needs (+clinical indications)<br><br>Category 2B<br>Potential cases in institutional accommodations based on what is most appropriate based on current resource needs (+clinical indications)	Regional and local adaptations are coordinated by Infection Control Practitioners based on what is most appropriate based on the current epidemiological situation and regional and local structures.
(12) 2020-05-08	Testing indication for nucleic acid detection at COVID-19<br><br>The indication refers to testing for laboratory analysis	Priority 1<br>Patients in need of inpatient care; inpatients at hospitals; individuals who have a 'disease picture' in which the treating physician assesses that early diagnosis is important for the treatment; residents in care and in institutions (+clinical indications)<br>Priority 2	Regional and local adaptations are coordinated by the region's crisis leadership and the Infection Control Practitioner based on what is most appropriate based on the

	with nucleic acid detection according to the priority groups.	Staff in health- and social care (+clinical indications)<br><br>**The groups described as priority 3 and 4 in the National Strategy will be included at a later stage. The work of defining the groups is currently underway.**	current epidemiological situation and regional and local structures.
(13) 2020-05-27	Testing indication for nucleic acid detection at COVID-19<br><br>The indication refers to testing for laboratory analysis with nucleic acid detection according to the priority groups.	Priority 1<br>Patients in need of inpatient care; inpatients at hospitals; individuals who have a 'disease picture' in which the treating physician assesses that early diagnosis is important for the treatment; residents in care and in institutions (+clinical indications)<br><br>Priority 2<br>Staff in health- and social care (+clinical indications)<br><br>Priority 3<br>Staff in services of importance for society (according to list published by the MSB) (+clinical indications)<br><br>**The group described as priority 4 in the National Strategy will be included at a later stage.**	Regional and local adaptations are coordinated by the region's crisis leadership and the Infection Control Practitioner based on what is most appropriate based on the current epidemiological situation and regional and local structures.
(14) 2020-06-17<br>(15) 2020-07-30	Testing indication for nucleic acid detection at COVID-19<br><br>The indication refers to testing for laboratory analysis with nucleic acid detection.	1) Individuals who show symptoms described for COVID-19<br>2) In case of contact tracing [1] and possible screening [2].<br><br>In a situation where capacity cannot meet the need, the following priorities are recommended: priority group 1, thereafter 2, thereafter 3.<br><br>[1] Contact tracing is done based on risk assessment and based on regional and local directives and may in some situations be relevant regardless of clinical picture.<br>[2] Possible screening prior to enrollment in eldercare/care institution or inpatient care based on risk assessment and based on regional and local directives and may in certain situations be independent of the clinical picture.	Regional and local adaptations are coordinated by the region's crisis leadership and the Infection Control Practitioner based on what is most appropriate based on the current epidemiological situation and regional and local structures.
(16) 2020-08-24	Testing indication for nucleic acid detection at COVID-19	1) Individuals who show symptoms described for COVID-19<br>2) In case of contact tracing and possible screening[1].	Regional and local adaptations are coordinated by the region's crisis leadership and the

		In a situation where capacity cannot meet the need, the following priorities are recommended: priority group 1, thereafter 2, thereafter 3.<br><br>The indication also specifies how to assess renewed testing in previously PCR-confirmed cases or individuals where IgG antibodies have been detected.<br><br>[1] Possible screening prior to enrollment in eldercare/care institution or inpatient care based on risk assessment and based on regional and local directives and may in certain situations be independent of the clinical picture.	Infection Control Practitioner based on what is most appropriate based on the current epidemiological situation and regional and local structures.
(17) 2020-11-26<br>(18) 2021-01-29	Indication for detection of ongoing COVID-19	Testing<br>• in case of symptoms of COVID-19<br>• In case of contact tracing<br>• In case of possible screening. For example prior to enrollment in eldercare/care institution or inpatient care based on risk assessment and based on regional and local directives and may in certain situations be independent of the clinical picture.	The region in consultation with the Infection Control Practitioner always has the possibility to make adjustments based on the regional situation

Source: Folkhälsomyndigheten. Testing recommendations 1-18.
