# Supplementary material for: COVID-19 Testing in Sweden During 2020–Split Responsibilities and Multi-Level Challenges
Source: Front Public Health. 2021 Nov 19;9:754861. doi: 10.3389/fpubh.2021.754861 (PMC8639858; doi:10.3389/fpubh.2021.754861)
Supplement: Supplementary file 2 [file Data_Sheet_2.PDF]

Table S2. Priorities and flows for testing and analysis

	Pre-analysis	Analysis	Post-analysis
Priority group 1	Contact with 1177, 112 and transport to health care for test	At lab according to local routine, and by a subcontractor if there is a lack of capacity	Test result and further handling based on this
	This group is already prioritized and it does not change in this strategy. The flow from pre-analysis to postal analysis is handled as before by healthcare within each region's assignment. In the event of a lack of capacity, a subcontractor can be hired for the analysis step.		
Priority group 2 (two possible tracks)	Ordered via occupational health care or referring doctor. Test within health care (local routine)	At lab according to local routine	Feedback to occupational health care or the referring doctor regarding test result
	Ordered via occupational health care or e-service. Distribution of test material for testing in the home or other dedicated site	At contracted laboratory	Feedback to occupational health care or the referring doctor regarding test result
	There is a lack of capacity. A complementary 'analytic flow' needs to be established and eventually replace the current 'flow'. It covers new actors for testing, logistics, data-transfer and analysis. Needs assessment is made by the employer.		
Priority group 3	Ordered via occupational health care or e-service. Distribution of test material for testing in the home or other dedicated site	At contracted laboratory	Feedback to occupational health care or referring doctor of test result
	Testing and analysis needs to be handles outside of the regions' ordinary assignment. New actors are needed for testing, logistics, data-transfer and analysis. Needs assessment is made by the employer.		
Priority group 4	Ordered via occupational health care or e-service. Distribution of test material for testing in the home or other dedicated site	At contracted laboratory	Feedback to occupational health care or referring doctor of test result
	At present, there is in principle no medical need for an individual to know whether the acute infection is COVID-19. However, there are societal benefits with reduced sick leave by employees who do not have COVID-19 being able to return to work more quickly in the event of a negative test result.		

Source: Folkhälsomyndigheten, 10-06-2020. Nationell strategi för utökad provtagning och laboratorieanalys av covid-19.
